# ZNF521/EBF1 axis regulates AKR1B1 to promote the proliferation, migration, and invasion of gastric cancer cells

**DOI:** 10.1002/kjm2.12624

**Published:** 2022-11-17

**Authors:** Yu Cheng, Yi‐Jiang Ni, Li‐Ming Tang

**Affiliations:** ^1^ Department of Gastrointestinal Surgery Changzhou No. 2 People's Hospital (Affiliated Hospital of Nanjing Medical University) Changzhou Jiangsu People's Republic of China

**Keywords:** AKR1B1, EBF1, gastric cancer, proliferation and invasiveness, ZNF521

## Abstract

Although the incidence and death rates of gastric cancer (GC) are decreasing, approximately one million new cases and 800,000 GC‐related deaths were reported worldwide in 2018. Currently, the oncogenesis of GC remains unclear, and the demand for novel treatment options are unmet. Here, we explored the role of aldo‐keto reductase family 1 member B (AKR1B1) in the progression of GC. The proliferation, migration, and invasion of GC cells were evaluated by CCK‐8 assay, wound healing assay, and transwell assay, respectively. The interaction between EBF transcription factor 1 (EBF1) and the promoter region of AKR1B1 was determined by luciferase reporter assay and chromatin immunoprecipitation (ChIP). Gene expression levels were measured by quantitative real‐time polymerase chain reaction (qRT‐PCR) and Western blotting assay. The expression of AKR1B1 was elevated in GC cells, resulting in increased cell proliferation, migration, and invasion. Meanwhile, EBF1 was a negative regulator of AKR1B1; its overexpression suppressed AKR1B1 expression and GC progression. Furthermore, knockdown of ZNF521 induced EBF1 expression, thus suppressing AKR1B1 expression and resulting in attenuated GC growth and invasiveness. Notably, knockdown of ZNF521 attenuated GC progression and was rescued by overexpression of AKR1B1. Our current study revealed a novel ZNF521/EBF1/AKR1B1 axis in GC and elaborated its important role in promoting GC progression, providing potential therapeutic targets for anti‐GC treatments.

## 1 INTRODUCTION

Among cancers, gastric cancer (GC) has a high mortality‐to‐incidence ratio. Age, diet, and stomach disease are the top risk factors for GC.^1^ GC is more prevalent in east Asia, with China accounting for 42.6% and 45.0% of GC cases and mortality worldwide, respectively.^2^, ^3^ Currently, surgery combined with chemotherapy can improve overall survival (OS) in GC^4^; OS in patients with advanced GC may be prolonged to 6–14 months following chemotherapy.^5^, ^6^ Many aspects regarding the pathogenesis of GC must be elucidated to enable the development of novel therapies.

Aldo‐keto reductase family 1 member B1 (AKR1B1) is a protein belonging to the aldehyde‐keto reductase superfamily that is encoded by the AKR1B1 gene in humans and is widely expressed in the kidneys, heart, brain, blood vessels, lungs, and liver.^7^ It reduces various aldehydes and ketones into alcohol and participates in the regulation of oxidative stress diseases, cell signal transduction, and cell proliferation.^8^, ^9^ AKR1B1 also plays important roles in malignancy; it activates epithelial‐mesenchymal transition to promote basal‐like breast cancer progression.^10^ Additionally, AKR1B1 expression is also associated with the prognosis of endometrial carcinomas.^11^ A recent study revealed that GC tissues showed higher AKR1B1 expression than paracancerous tissues, and that GC patients with high levels of AKR1B1 have poorer prognoses.^12^, ^13^ However, the role of AKR1B1 in GC tumorigenesis and the underlying mechanisms involved remain unexplored.

Transcription factor early B‐cell factor 1 (EBF1) controls the expression of essential genes for B cell differentiation and function^14^; however, it is also implicated in oncogenesis. EBF1 is downregulated in colorectal cancers and exerts a tumor suppressing effect by modulating p53 signaling.^15^, ^16^ EBF1 represses the transcription of telomerase catalytic subunit and suppresses the progression of GC.^17^ Exploiting the online bioinformatic tool JASPAR (http://jaspar.genereg.net/), we found potential binding sites in the upstream promoter region of AKR1B1. Besides, the ENCyclopedia of DNA Elements (ENCODE) study identified AKR1B1 as a transcriptional target of EBF1.^18^, ^19^ Thus, we hypothesized that EBF1 may regulate the transcription of AKR1B1 in GC.

Zinc finger protein 521 (ZNF521), which is a transcription co‐factor, has a variety of functions in multiple cells, including hematopoietic, osteo‐adipogenic, neural progenitor, and cancer cells. ZNF521 promotes GC cancer cell progression^20^ and is a repressor of EBF1.^21^, ^22^ Therefore, we hypothesized that ZNF521 may promote cancer progression via suppression of EBF1 activity in GC.

Here, we evaluated the expression of AKR1B1 in non‐cancerous gastric epithelial and GC cells and assessed its role in regulating GC progression. We further investigated the interaction between EBF1 and the AKR1B1 promoter. Finally, we explored the regulation of EBF1 by ZNF521.

## 2 MATERIALS AND METHODS

### 2.1 Cell culture

Human gastric epithelial cell line GES‐1 and GC cell lines AGS and NCI‐N87 from Procell Life Science Technology Co., Ltd. were cultured in Roswell Park Memorial Institute 1640 medium (RPMI 1640, Cat. #11875093, Gibco) supplemented with 10% fetal bovine serum (FBS) and 1% of penicillin–streptomycin‐glutamine solution (Cat. #10378016, Gibco) at 37°C in an atmosphere containing 5% CO_2_.

### 2.2 Plasmid construction and transfection

The protein encoding region of human AKR1B1 and EBF1 were cloned and inserted into pcDNA3.1 vector (Addgene). Human AKR1B1‐, ZNF521‐, and EBF1‐targeting shRNAs were purchased from GenePharma. Cells were cultured in a six‐well plate overnight to reach 80% confluency before plasmid transfection with Lipofectamine 3000 (Cat. #L3000015, Invitrogen). Briefly, 4 μl of Lipofectamine 3000 and 2.5 μg of plasmid were separately diluted in 125 μl of Opti‐MEM Medium (Cat. #31985062, Gibco), and the diluted DNA solution was added into the Lipofectamine 3000 solution and mixed well followed by 15 min of incubation at room temperature. Finally, the DNA–lipid transfection complex was added into cells and incubated for 48 h before further experimentation.

### 2.3 Wound healing assay

Cells were cultured to form a 70%–80% confluent monolayer in a six‐well plate. The single layer of cells was scraped by a pipette tip, and the floating cells were removed along with PBS followed by replenishment with 1.5 ml of serum‐free medium. Cells were imaged at 0 h under the microscope and imaged again after culture for an additional 24 h.

### 2.4 Cell counting kit‐8 (CCK8) assay

Cell viability was determined by cell counting kit (CCK‐8) assay from Abcam (Cat. #ab228554). A total of 4 × 10^3^ cells were seeded in a 96‐well plate for treatment. At the endpoint, 10 μl of CCK‐8 solution was added, and the solution was incubated for an additional 3 h in the dark. The absorbance at 450 nm (OD_450_) was then measured.

### 2.5 Transwell assay

A total of 50,000 cells in 0.5 ml of FBS‐free medium were seeded in extracellular matrix protein‐coated chambers (Cat. #354480, Corning). Then, 0.5 ml of complete medium was added into the bottom well as an attractant, and the chambers were washed with PBS 24 h later. The samples were fixed in 4% paraformaldehyde and stained with 1% crystal violet. Cells were then counted and imaged under a microscope.

### 2.6 Luciferase reporter assay

The promoter region and corresponding mutant region of AKR1B1 was inserted into the pGL3 luciferase reporter vector (Promega). A wild type (WT) or mutant (MUT) AKR1B1 luciferase reporter gene vector was co‐transferred with oe‐EBF1 into cells by Lipofectamine 3000 (Invitrogen) as previously described. A dual‐luciferase reporter assay system (Cat. #E1910, Promega) was used to measure luciferase activity 48 h later by a SpectraMax microplate reader. Briefly, cells were co‐transfected with EBF1 luciferase with WT or MUT AKR1B1 luciferase reporter for 48 h in a 96‐well plate. Wells with medium alone served as blank controls. Then, 20 μl of cell lysate was mixed with 100 μl of LAR II reagent, and the firefly luciferase activity was recorded. Subsequently, 100 μl of stop and glo reagent was added, and *Renilla* luciferase activity was measured. *Renilla* luciferase activity was then normalized against the firefly luciferase activity.

### 2.7 Chromatin immunoprecipitation (ChIP)

Chromatin immunoprecipitation (ChIP) was performed using the ChIP kit (Cat. #ab500, Abcam). Protein–DNA complexes was crosslinked in 0.75% formaldehyde for 15 min at room temperature and quenched with 125 mM glycine. Cells were lysed in a lysis buffer with protease inhibitor cocktails, sonicated, and centrifuged. The supernatant containing 50 μg of DNA complex was incubated with 5 μg of anti‐EBF1 antibody (Cat. #50752, Cell Signaling Technology) for 1 h at 4°C, and the DNA–protein complexes were pulled down by protein A/G agarose beads (Thermo Scientific). The promoter of AKR1B1 was checked by quantitative real‐time polymerase chain reaction (qRT‐PCR).

### 2.8 Western blotting analysis

Cells were lysed in RIPA buffer with protease inhibitor cocktails. Proteins were separated by 10% SDS‐PAGE and transferred onto polyvinylidene difluoride membrane. The blot was blocked in TBST buffer containing 5% BSA and incubated for at least 4 h at 4°C with the primary antibody. Horseradish peroxidase‐conjugated secondary antibodies (α‐mouse: Cat. #115‐035‐164, or α‐rabbit: Cat. #111‐035‐003, Jackson ImmunoResearch) were used for blot developing with enhanced chemiluminescence kits (Cat. #1705060 S, BIO‐RAD). Images were captured using a ChemicDoc XRS system (Bio‐Rad). Relative protein levels were calculated using ImageJ software. The following antibodies were used for Western blotting experiments: GAPDH (Cat. #437000, Invitrogen), AKR1B1 (Cat. #PA5‐117777, Invitrogen), EBF1 (Cat. #50752, Cell Signaling Technology), ZNF521 (Cat. #PA5‐34388, Invitrogen).

## 3 QUANTITATIVE REVERSE‐TRANSCRIPTION POLYMERASE CHAIN REACTION

Total RNA was extracted by RNeasy Mini Kit (Cat. #74104, QIAGEN, Hilden). cDNA was synthesized immediately with High‐Capacity cDNA Reverse Transcription Kit (Cat. #4368814, Applied Biosystems). qRT‐PCR was performed using SsoAdvanced Universal SYBR Green Supermix (Cat. #1725270, BIO‐RAD). β‐Actin was used as the reference gene. The following primers were used to determine the indicated gene expression.

AKR1B1‐F: TGCCACCCATATCTCACTCA

AKR1B1‐R: TGTCACAGACTTGGGGATCA

EBF1‐F: CATGCGGAGATTCCAGGTCG

EBF1‐R: AAGAGGGCGTACCTTCCGA

ZNF521‐F: CCCAGTCGGATGAGAAGAAGA

ZNF521‐R: ATCCCTTCGAAGCTGTGCTC

β‐actin‐F: TGGCACCACACCTTCTACAA

β‐actin‐R: CCAGAGGCGTACAGGGATAG

### 3.1 Statistics

The experiment was repeated at least three times. Data were shown as mean ± standard deviation. Student's *t* test, one‐ or two‐way analysis of variance (with the following Tukey post hoc test) were used for statistical analysis with GraphPad Prism software (GraphPad Software Inc.). *p* < 0.05 was considered statistically significant.

## 4 RESULTS

### 4.1 Loss of AKR1B1 attenuates the proliferation, migration, and invasion of gastric cancer cells

**FIGURE 1:**
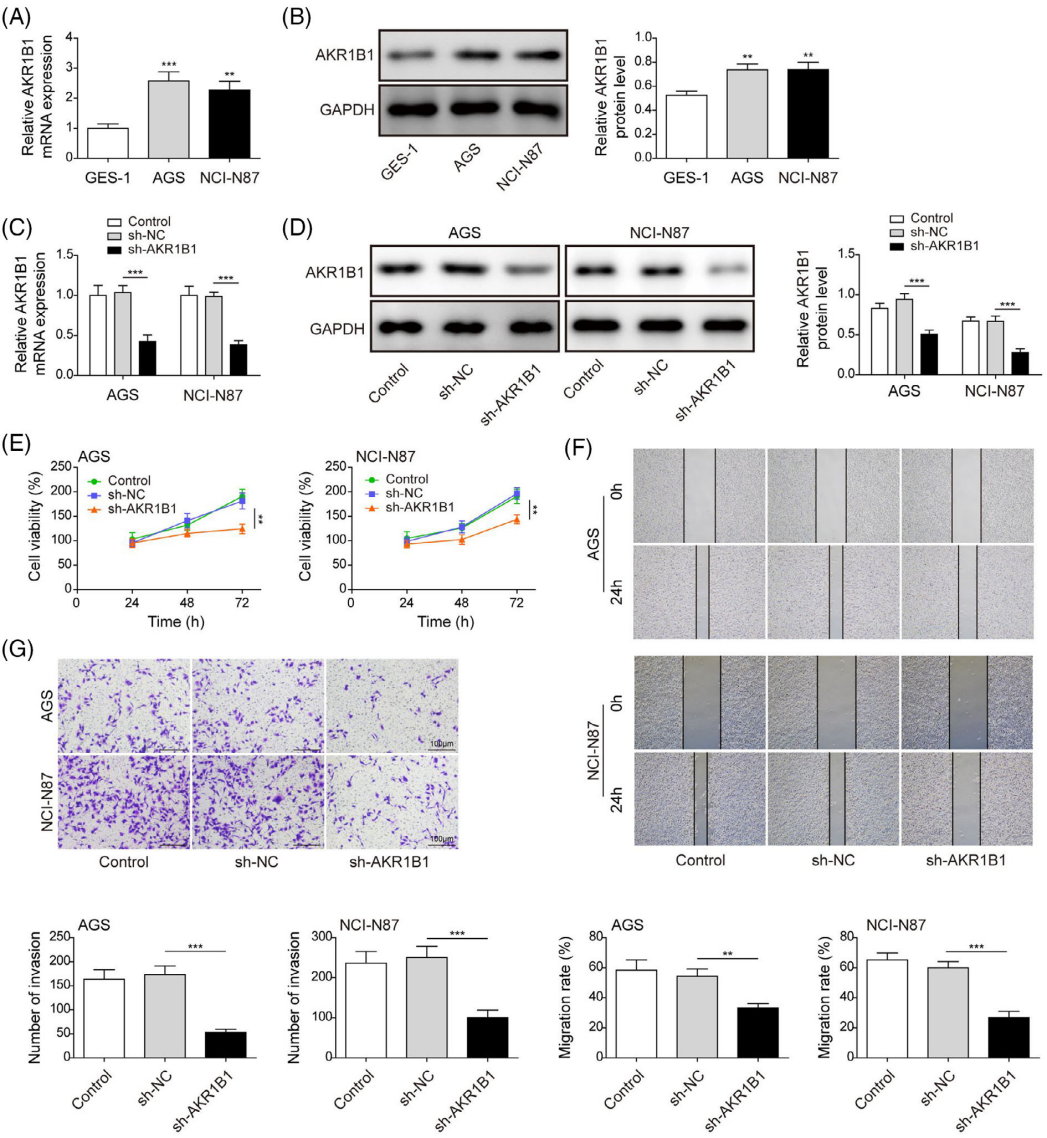
Loss of AKR1B1 attenuates the proliferation, migration, and invasion of gastric cancer cells. (A) and (B) AKR1B1 levels in GES‐1, AGS, and NCI‐N87 were determined by qRT‐PCR and immunoblotting assay. (C) and (D) qRT‐PCR and immunoblotting assay for mRNA and protein level of AKR1B1 in AGS and NCI‐N87 expressing sh‐NC or sh‐AKR1B1. (E) CCK‐8 cell proliferation assay in cells expressing sh‐NC or sh‐AKR1B1. (F) Wound healing assay for the migration of cancer cells expressing sh‐NC or sh‐AKR1B1. (G) Transwell assay for invasion of cancer cells expressing sh‐NC or sh‐AKR1B1. Data were presented as mean ± SD. *N* = 3. *p < 0.05, **p < 0.01, ***p < 0.001. qRT‐PCR, quantitative real‐time polymerase chain reaction

Levels of AKR1B1 in non‐cancerous gastric epithelial cells (GES‐1) and GC cells (AGS and NCI‐N87) were determined and were revealed to be significantly higher in cancer cells than in normal cells (Figure 1A,B). Therefore, AKR1B1 was knocked down in GC cells to evaluate its role in cell proliferation. AKR1B1‐targeting shRNA efficiently downregulated the mRNA and protein levels of AKR1B1 in GC cells (Figure 1C,D), resulting in suppression of proliferation (Figure 1 E). Similarly, the migration and invasion of cells with lower AKR1B1 expression were inhibited (Figure 1F,G). Our data demonstrated that knockdown of AKR1B1 plays essential roles in suppressing GC cell proliferation, migration, and invasion.

### 4.2 EBF1 negatively regulates AKR1B1 expression

**FIGURE 2:**
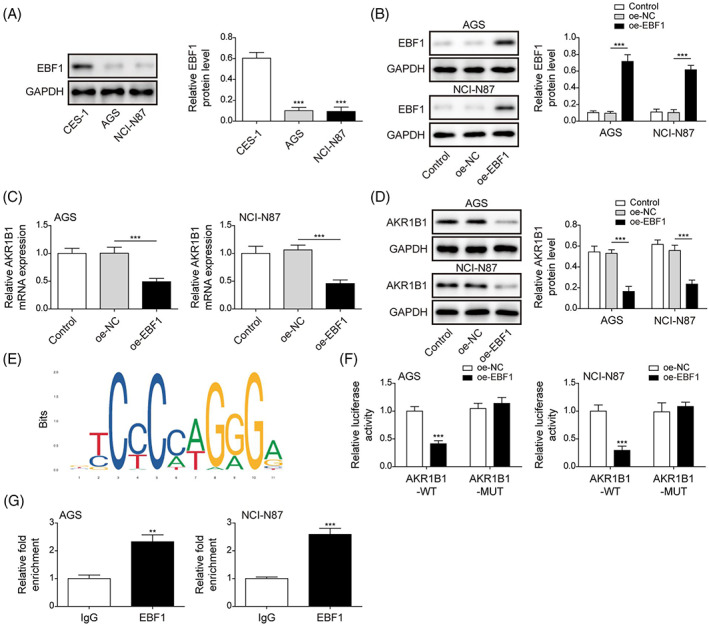
EBF1 negatively regulates AKR1B1 expression. (A) EBF1 levels in GES‐1, AGS, and NCI‐N87 were determined by immunoblotting assay. (B) Western blot assay for protein level of EBF1 in AGS and NCI‐N87 expressing control plasmid or EBF1. (C) and (D) qRT‐PCR and Western blot assay for mRNA and protein level of AKR1B1 in AGS and NCI‐N87 with EBF1 overexpression. (E) Bioinformation websites JASPAR was used to predict binding sites of EBF1 and the promoter region of AKR1B1. (F) Luciferase assay in cells co‐transfected with EBF1 and WT or MUT AKR1B1 promoter luciferase reporter. (G) ChIP assay for the interaction between EBF1 and the promoter region of AKR1B1. Data were presented as mean ± SD. *N* = 3. *p < 0.05, **p < 0.01, ***p < 0.001. MUT, mutant; qRT‐PCR, quantitative real‐time polymerase chain reaction; WT, wild type

We found that EBF1 expression was decreased in GC cells (Figure 2A). As EBF1 was hypothesized to regulate AKR1B1 expression, we overexpressed EBF1 in AGS and NCI‐N87 cells. EBF1 was successfully overexpressed in both cell lines (Figure 2B), and its overexpression led to the downregulation of AKR1B1 at both mRNA and protein levels (Figure 2C,D). Using the online bioinformatic tool JASPAR (http://jaspar.genereg.net/), we found several potential EBF1 binding sites in the promoter region of AKR1B1 (Figure 2E). Overexpressed EBF1 dramatically reduced the luciferase activity of AKR1B1‐WT but not of the AKR1B1‐MUT luciferase reporter, demonstrating the direct targeting of AKR1B1 by EBF1 (Figure 2F). Additionally, ChIP assay revealed EBF1 binding to the AKR1B1 promoter region (Figure 2G). Collectively, these data showed that EBF1 binds to AKR1B1 and suppresses its transcription.

### 4.3 Overexpression of AKR1B1 rescues EBF1 overexpression‐mediated inhibition of gastric cancer cell progression

**FIGURE 3:**
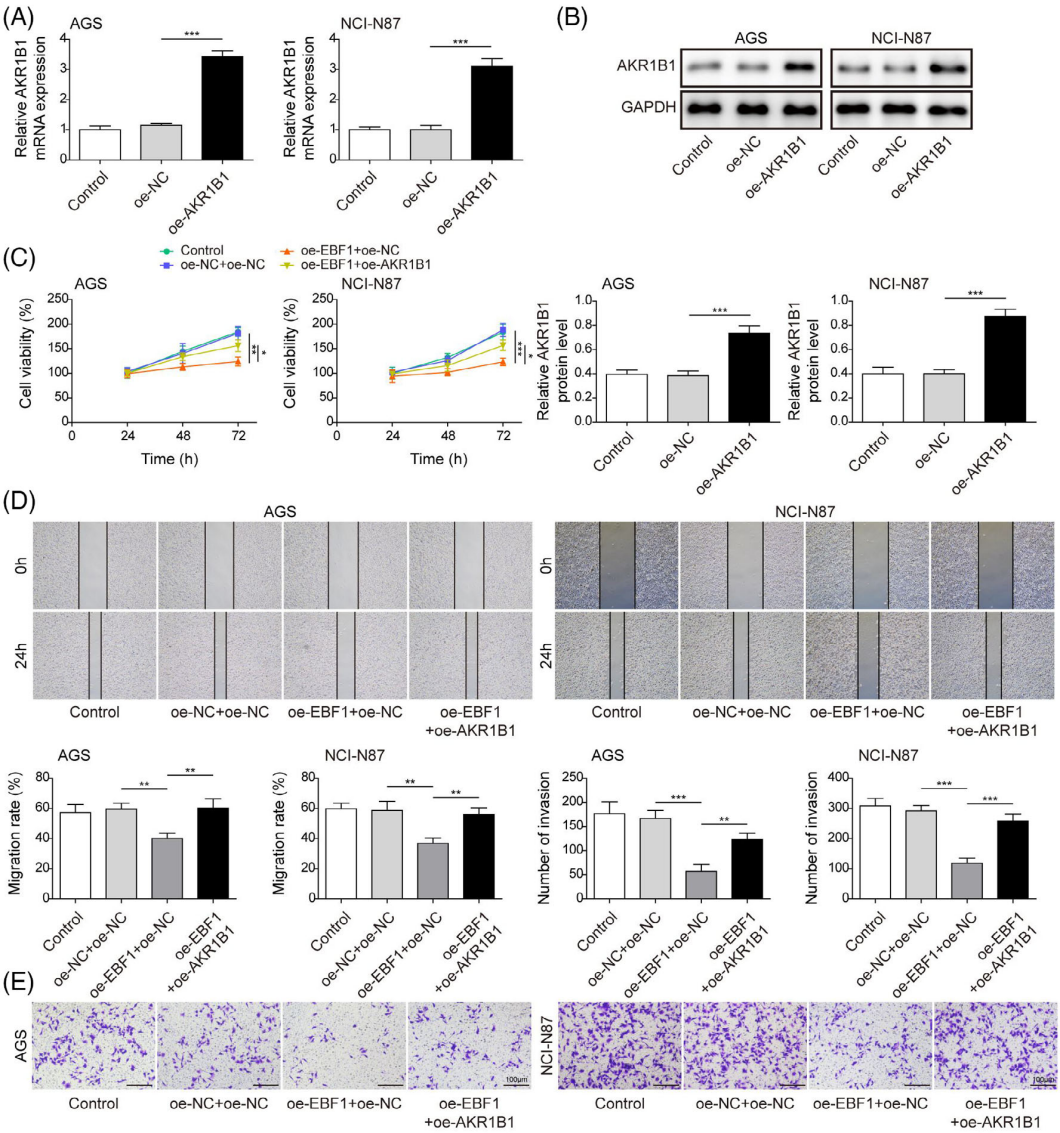
Overexpression of AKR1B1 rescues EBF1 overexpression‐mediated inhibition of gastric cancer cell progression. (A) and (B) qRT‐PCR and Western blot assay for mRNA and protein level of AKR1B1 in AGS and NCI‐N87. (C) CCK‐8 cell proliferation assay in cells overexpressed EBF1 with or without AKR1B1. (D) Wound healing assay for the migration of cancer cells overexpressed EBF1 with or without AKR1B1. (E) Transwell assay for invasion of cancer cells overexpressed EBF1 with or without AKR1B1. Data were presented as mean ± SD. *N* = 3. *p < 0.05, **p < 0.01, ***p < 0.001.

To test if AKR1B1 overexpression attenuates EBF1‐mediated suppression of GC cell progression, overexpressed AKR1B1 was introduced into GC cells, resulting in upregulation of mRNA and protein levels of AKR1B1 in both cells (Figure 3A,B). Overexpression of EBF1 inhibited cancer cell proliferation, but overexpression of AKR1B1 reversed the inhibitory effect of EBF1 on cell growth (Figure 3C). Additionally, EBF1 suppressed the migration and invasion of both cell lines, which was rescued by overexpression of AKR1B1 (Figure 3D,E). These data suggested that EBF1 exerts a tumor suppressor effect by inhibiting AKR1B1.

### 4.4 Loss of ZNF521 upregulates EBF1 to suppress AKR1B1 and attenuate the proliferation, migration, and invasion of gastric cancer cells

**FIGURE 4:**
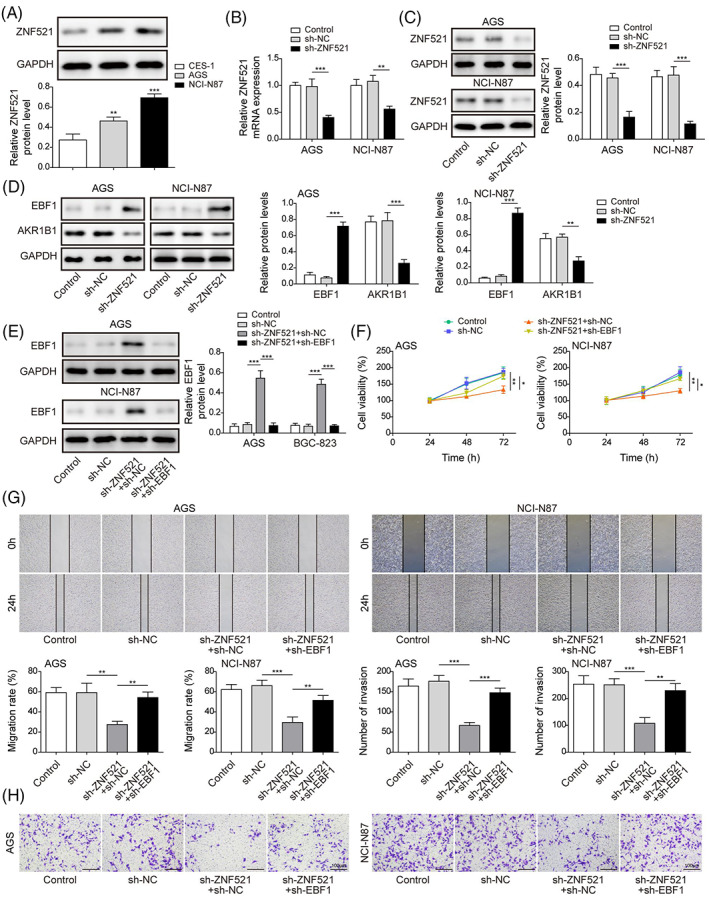
Loss of ZNF521 upregulates EBF1 to suppress AKR1B1 and attenuates the proliferation, migration, and invasion of gastric cancer cells. (A) ZNF521 levels in GES‐1, AGS, and NCI‐N87 were determined by immunoblotting assay. (B) and (C) qRT‐PCR and Western blot assay for mRNA and protein level of ZNF521 in AGS and NCI‐N87 cells expressing ZNF521‐targeting shRNA (sh‐ZNF521) or controls. (D) Western blot assay for protein level of EBF1 and AKR1B1 in AGS and NCI‐N87 cells with or without sh‐ZNF521. (E) Western blot assay for protein level of EBF1 in AGS and NCI‐N87 cells expressing sh‐ZNF521 with or without sh‐EBF1. (F) CCK‐8 cell proliferation assay in cells expressing sh‐ZNF521 with or without sh‐EBF1. (G) Wound healing assay for the migration of cancer cells expressing sh‐ZNF521 with or without sh‐EBF1. (H) Transwell assay for invasion of cancer cells expressing sh‐ZNF521 with or without sh‐EBF1. Data were presented as mean ± SD. *N* = 3. *p < 0.05, **p < 0.01, ***p < 0.001.

We hypothesized that ZNF521 plays a role in GC by regulating EBF1. First, the expression of ZNF521 in GC cells was detected, and the results showed that ZNF521 was highly expressed in GC cells (Figure 4A). By knocking down ZNF521, the mRNA and protein levels of ZNF521 were significantly inhibited in AGS and NCI‐N87 cells (Figure 4B,C). Knockdown of ZNF521 enhanced EBF1 expression, which subsequently reduced AKR1B1 expression (Figure 4D). We further knocked down EBF1 in cells expressing sh‐ZNF521 (Figure 4E). Loss of ZNF521 attenuated the cell viability of AGS and NCI‐N87 cells, while further inhibition of EBF1 increased cell viability (Figure 4F). EBF1 knockdown also reversed the migratory and invasive abilities suppressed by ZNF521 knockdown (Figure 4G,H). These data demonstrated that as an upstream regulator of EBF1, suppression of ZNF521 attenuates GC progression by inducing EBF1expression.

### 4.5 AKR1B1 overexpression rescues gastric cancer progression attenuated by ZNF521 knockdown

**FIGURE 5:**
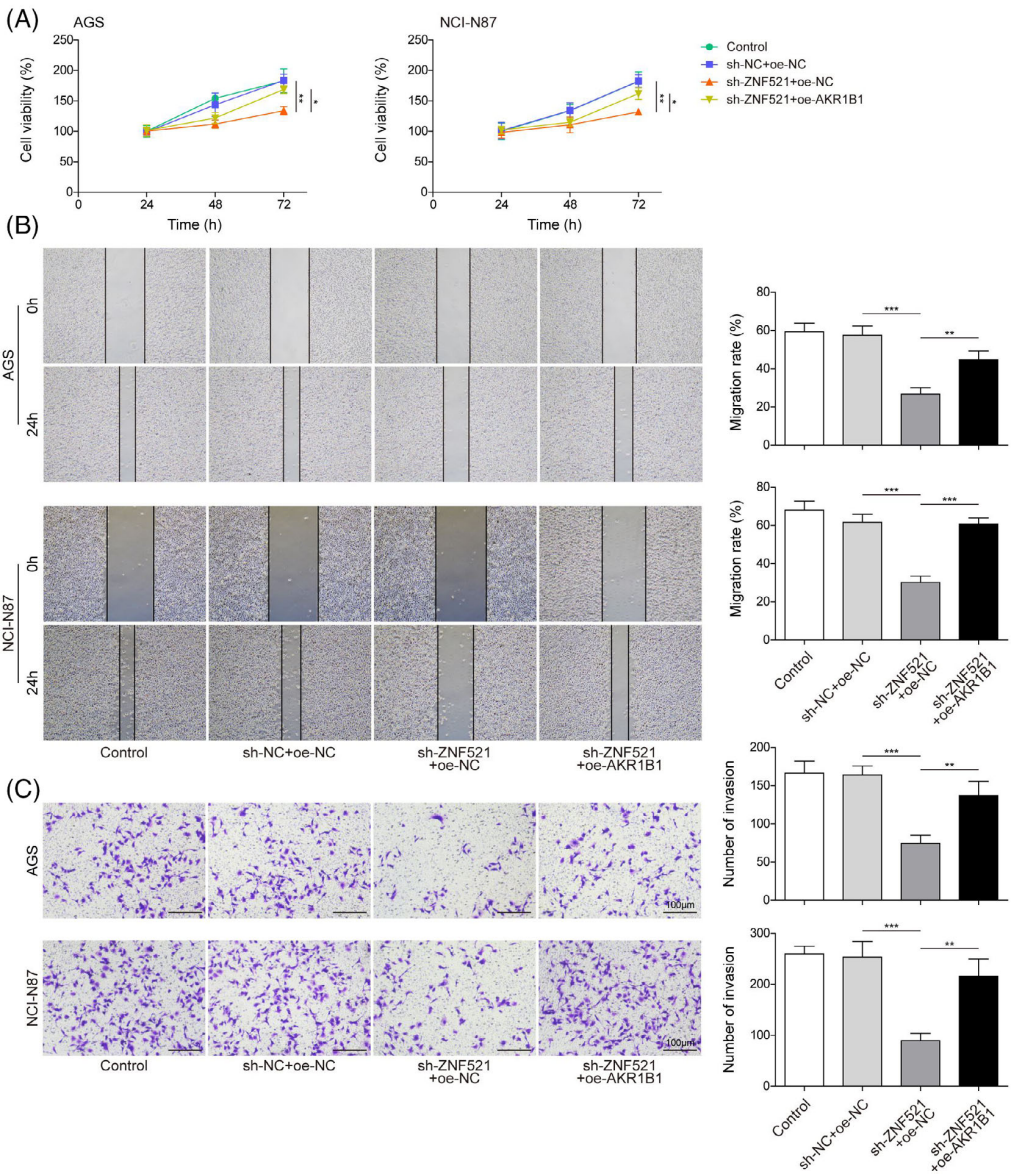
AKR1B1 overexpression rescues gastric cancer progression attenuated by ZNF521 knockdown. (A) CCK‐8 cell proliferation assay for cells expressing sh‐ZNF521 with or without oe‐AKR1B1. (B) Wound healing assay for the migration of cancer cells expressing sh‐ZNF521 with/without oe‐AKR1B1. (C) Transwell assay for invasion of cancer cells expressing sh‐ZNF521 with/without oe‐AKR1B1. Data were presented as mean ± SD. *N* = 3. *p < 0.05, **p < 0.01, ***p < 0.001

To explore if AKR1B1 can rescue attenuated GC progression upon loss off ZNF521, we overexpressed AKR1B1 in AGS and NCI‐N87 cells expressing ZNF521‐targeting shRNA. Knockdown of ZNF521 inhibited GC cell growth, but this effect was reversed by AKR1B1 overexpression (Figure 5A). In addition, the inhibitory effects of ZNF521 knockdown on migration and invasion were attenuated after AKR1B1 overexpression. (Figure 5B,C). These results revealed that ZNF521 regulates GC progression through AKR1B1.

## 5 DISCUSSION

**FIGURE 6:**
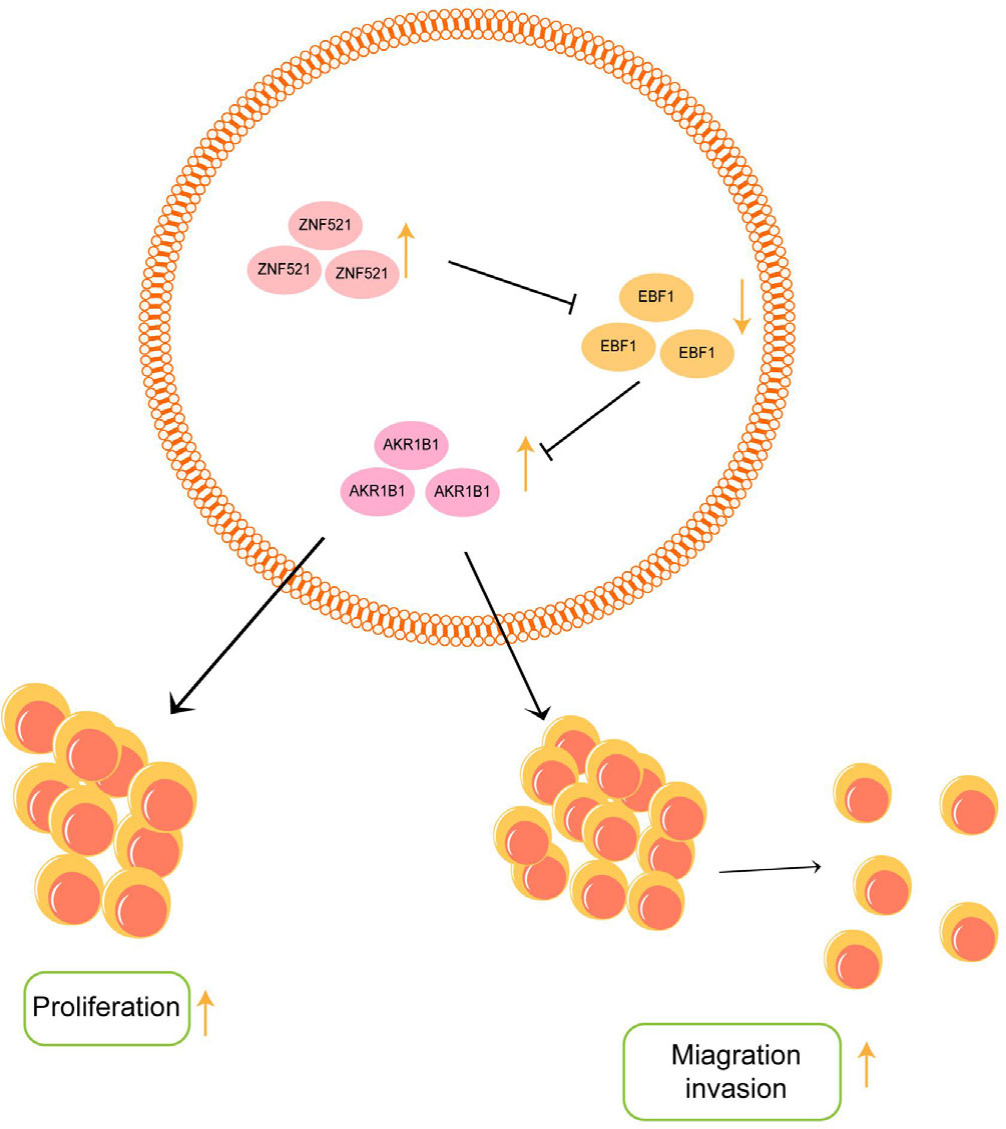
AKR1B1 is regulated by the ZNF521/EBF1 axis in the proliferation, migration, and invasion of gastric cancer. As transcription factors, both ZNF521 and EBF1 negatively regulated the expression of downstream genes and that the high expression of ZNF521 reduced the transcription of EBF1, thereby promoting the transcription of AKR1B1. It promoted the proliferation, migration, and invasion of gastric cancer cells

GC has one of the highest mortality rates among cancers, particularly in east Asia. Additionally, the lack of information regarding the pathogenesis of impedes the development of effective treatments. Our current study identified the ZNF521/EBF1/AKR1B1 axis, which is hyperactivated in GC and plays important role in promoting cancer cell growth, migration, and invasion. By binding with EBF1, ZNF521 antagonized its transcription repressor function on the expression of AKR1B1, liberating the tumor‐promoting activity of AKR1B1. Thus, targeting this tumor‐promoting axis may be a potential anti‐GC strategy (Figure 6).

AKR1B1 is implicated in the progression of multiple type of cancers, but the underlying mechanisms remains elusive, particularly in GC. Recent studies revealed that levels of AKR1B1 are higher in GC tissues than in paracancerous tissues, and its elevation is correlated with worse outcomes in GC patients.^12^, ^13^ Consistently, we found elevated AKR1B1 expression in GC cell lines, and that loss of AKR1B1 led to compromised cell growth and invasiveness. To better understand the transcriptional regulation of AKR1B1, we exploited bioinformatic analysis and identified EBF1 as a potential regulator of AKR1B1. EBF1 is a potential tumor suppressor in malignancies, including breast cancer^23^; genomic deletion of EBF1 occurs in approximately 19% of breast cancer cell lines.^24^ Other studies demonstrated that EBF1 is epigenetically silenced in GC, probably through promoter hypermethylation, while the somatic mutation rate of *EBF1* in GC is around 3.1%.^17^ Some of these mutations disrupts the suppressive activity of EBF1 on cell proliferation.^17^ Although the role of EBF1 as a transcription factor is well studied,^14^ studies on EBF1 in malignancies is scarce. In addition to its role in modulating p53 signaling in colorectal cancer,^15^, ^16^ we identified AKR1B1 as a novel target of EBF1 in GC. Loss of AKR1B1 attenuated the progression of GC, which is similarly achieved by overexpression of EBF1, while overexpression of AKR1B1 weakened the tumor suppressor activity of EBF1. Collectively, these data demonstrated that AKR1B1 is essential for GC growth and invasion, while EBF1, as a negative regulator, represses the expression of AKR1B1, thus inhibiting GC progression.

Little is known about the role of ZNF521 in GC. Early studies found that ZNF521 was upregulated in GC, which promoted its development.^20^ Additionally, ZNF521 expression was associated with GC TNM stage, tumor size, and local lymph node metastasis.^20^ The authors also revealed that miRNA‐204‐5p, which is commonly downregulated in cancers, is a negative regulator of ZNF521 expression.^20^ MiRNA‐204‐5p exerts tumor suppressor activity in a variety of cancers, including GC wherein it targets USP47, RAB22A, and HER2.^25^, ^26^ ZNF521 is an antagonist of EBF1,^21^, ^22^ but it is unknown if ZNF521 also actively antagonizes EBF1 in GC. As expected, we observed increased expression of EBF1 upon ZNF521 knockdown, suggesting that knockdown of ZNF521 may be involved in GC tumorigenesis by promoting EBF1. Moreover, ZNF521 knockdown‐mediated elevation of EBF1 expression resulted in downregulation of AKR1B1 and subsequent inhibition of cancer cell proliferation, migration, and invasion. More importantly, knockdown of ZNF521 failed to inhibit GC progression in the presence of AKR1B1 overexpression, demonstrating that AKR1B1 is essential in the ZNF521‐mediated tumor‐promoting activity of ZNF521.

Collectively, our current study evaluated the function of AKR1B1 and thoroughly investigated its transcriptional regulation in GC. Ultimately, we identified the ZNF521/EBF1/AKR1B1 axis, which is highly active in GC and promotes its development, providing novel insights in GC pathogenesis and potential therapeutic targets. Further investigation is warranted to explore these potential targets for anti‐GC treatment.

## CONFLICT OF INTEREST

The authors declare no conflict of interest.
